# Species diversity and risk factors of gastrointestinal nematodes in smallholder dairy calves in Kenya

**DOI:** 10.3389/fvets.2025.1588350

**Published:** 2025-08-12

**Authors:** Sylvia Cheptoo, Erhan Yalcindag, Lina González Gordon, Benson Rukwaro, Joseph Samuel Kimatu, Joseph Wasonga, Benedict E. Karani, Gideon Ndambuki, Susan Migeni, Jesse Kagai, Linus Eric Kiprotich, Nelson Saya, Deepali Vasoya, Getrude Nangekhe, Justus Onguso, Grace Mungai, Barend Mark Bronsvoort, Elizabeth Anne Jessie Cook

**Affiliations:** ^1^Jomo Kenyatta University of Agriculture and Technology (JKUAT), Nairobi, Kenya; ^2^Centre for Tropical Livestock Genetics and Health (CTLGH), International Livestock Research Institute (ILRI), Nairobi, Kenya; ^3^Centre for Tropical Livestock Genetics and Health (CTLGH), Roslin Institute, University of Edinburgh, Easter Bush Campus, Edinburgh, United Kingdom; ^4^Department of Veterinary Pathology, Microbiology and Parasitology, The University of Nairobi, Nairobi, Kenya; ^5^Department of Public Health, Pharmacology and Toxicology, The University of Nairobi, Nairobi, Kenya; ^6^Department of Community Health, Amref International University (AMIU), Nairobi, Kenya; ^7^Mazingira Centre, International Livestock Research Institute (ILRI), Nairobi, Kenya; ^8^UK Dementia Research Institute at University of Edinburgh, Edinburgh, United Kingdom

**Keywords:** gastrointestinal nematodes, deep amplicon sequencing, risk factor, co-infections, dairy calves, Kenya

## Abstract

Gastrointestinal nematodes (GIN) are of major concern in dairy farming, particularly in smallholder systems, because of their impact on the health of the calves and later on their productivity. These infections often occur as co-infections, which can complicate their prevention and treatment. The aim of this study was to conduct fecal egg counts (FEC), genetically identify GIN species, assess species diversity, and identify associated risk factors for GIN infections in dairy calves. Fecal samples were collected from 532 dairy calves across 289 small holder dairy farms. Species identification was achieved through deep amplicon sequencing of the Internal Transcribed Spacer-2 rDNA locus (ITS-2) of first-stage larvae (L1). The mean eggs per gram (EPG) was 62.0 ± 93.0. Most of the calves 64.2% had low-intensity infections (<50 EPG), 28.6% had medium-intensity infections (50–200 EPG), and 7.2% had high-intensity infections (>200 EPG). Next Generation Sequencing analysis identified nine GIN species, with *Cooperia punctata* (27.8%), *Haemonchus placei* (26.3%), and *Haemonchus contortus* (23.6%) being the most prevalent. Co-infections were common, accounting for 69.5% of all infections, with two (40.1%), three (26.9%), and four-species combinations (19.8%) predominating. Male calves showed a significant association with both increased FEC and smaller heart girth, while FEC decreased with age. *H. placei* and *C. punctata* were associated with increased FEC, whereas *Ostertagia ostertagi* (14.5%) and *Trichostrongylus colubriformis* (8.0%) were associated with decreased heart girth. Calves managed under pasture systems had higher odds of co-infection. This study reveals that GIN infections are highly prevalent in dairy calves, with co-infections being common, and that GIN burden is significantly influenced by calf age, sex, and management system. The Nemabiome tool offers a promising approach to assessing GIN burden and guiding the selection of anthelmintic protocols as part of sustainable farming strategies in tropical regions.

## 1 Introduction

Gastrointestinal nematodes (GIN) infections are a global concern for livestock, particularly in pasture-based ruminants (1, 2). These infections often result in morbidity and productivity losses (3). First-season grazing calves are predominantly affected (4), showing clinical signs such as diarrhea, stunted growth and weight loss (5). Heavily parasitized calves suffer extensive damage to the intestinal lining and can develop hematological changes including severe anemia and hypoproteinaemia, which can lead to mortality (6). However, in dairy cattle, the most important impact of GIN infections are the subclinical, chronic production loses such as decreased milk production, with an estimated loss of 1.4 liter per cow per day (7), as well as lower milk protein content (8).

GIN exist in complex communities comprising several co-infecting species (9, 10), and co-infection is widespread in cattle globally (11, 12). Co-infections are associated with more severe pathology than single infections and can influence the host immune response by increasing susceptibility to other microparasitic infections, as well as changing the transmission patterns and dynamics of these infections within populations (13–15). Accurate species identification of co-infections is essential for guiding treatment decisions, particularly considering the rapid development of resistance to available anthelminthic drugs (16–18).

Conventional diagnostic methods rely on coproscopy using microscopic examination (19). GIN eggs have similar morphology, making it necessary to culture them to identify the third-stage larvae (L3) based on their distinct morphological features (20). However, some GIN species L3 have overlapping morphological features, making their identification harder (21). This has led to the development of molecular assays to allow accurate identification of GIN species in ruminants (22). Such assays include real-time polymerase chain reaction (qPCR), droplet digital PCR (ddPCR), PCR-linked Restriction Fragment Length Polymorphism (RFLP), multiplex PCR and multiplexed-tandem PCR (23–26). Although these methods can identify species-level infections, they are limited to predetermined species, potentially overlooking novel or non-targeted species (12).

A novel deep amplicon sequencing method, termed “Nemabiome,” was recently developed to examine nematode populations using the ITS-2 rDNA region. The locus of choice reliably differentiates between Clade V nematodes to the species level, most of which infect cattle (10). Within this clade is the *Trichostrongyloidea* family, including important genera such as *Haemonchus, Ostertagia, Cooperia*, *Trichostrongylus*, and *Teladorsagia* (27, 28). L3 have been commonly used for Nemabiome analysis (10, 12), however first-stage larvae (L1) are becoming an increasingly popular alternative due to improved larval recovery, a more labor-friendly process, and significantly shorter turnaround time of approximately 48 h compared to 21 days for L3 (3, 21).

The Nemabiome technique has been effectively used to identify GIN infections across various species and geographical areas, including beef cattle (12, 29) and heifers in Canada (30), sheep in Sweden and the United Kingdom (21, 31), wildlife in France (32), and horses from Thailand, United States, and Canada (33, 34). However, in Sub-Saharan Africa, studies on GIN infection among dairy calves are scarce, and rely on conventional methods (7, 35–43). To address this gap, this study investigates the occurrence and genetic diversity of GIN species using deep amplicon sequencing and explores the risk factors associated with GIN co-infections among smallholder dairy calves in a key dairy farming region in Kenya.

## 2 Materials and methods

### 2.1 Ethics statement

Scientific and ethical approval for the study was obtained from the International Livestock Research Institute (ILRI) Animal Care and Use Committee (ILRI-IACUC2023-10), Institutional Research Ethics Committee (ILRI-IREC2023-44) and The National Commission for Science and Technology (NACOSTI). Written consent forms were signed by farmers willing to take part in the study. Sampling did not pose any long-term risk for the calves and was conducted by qualified veterinary professionals.

### 2.2 Study setting

**Figure 1:**
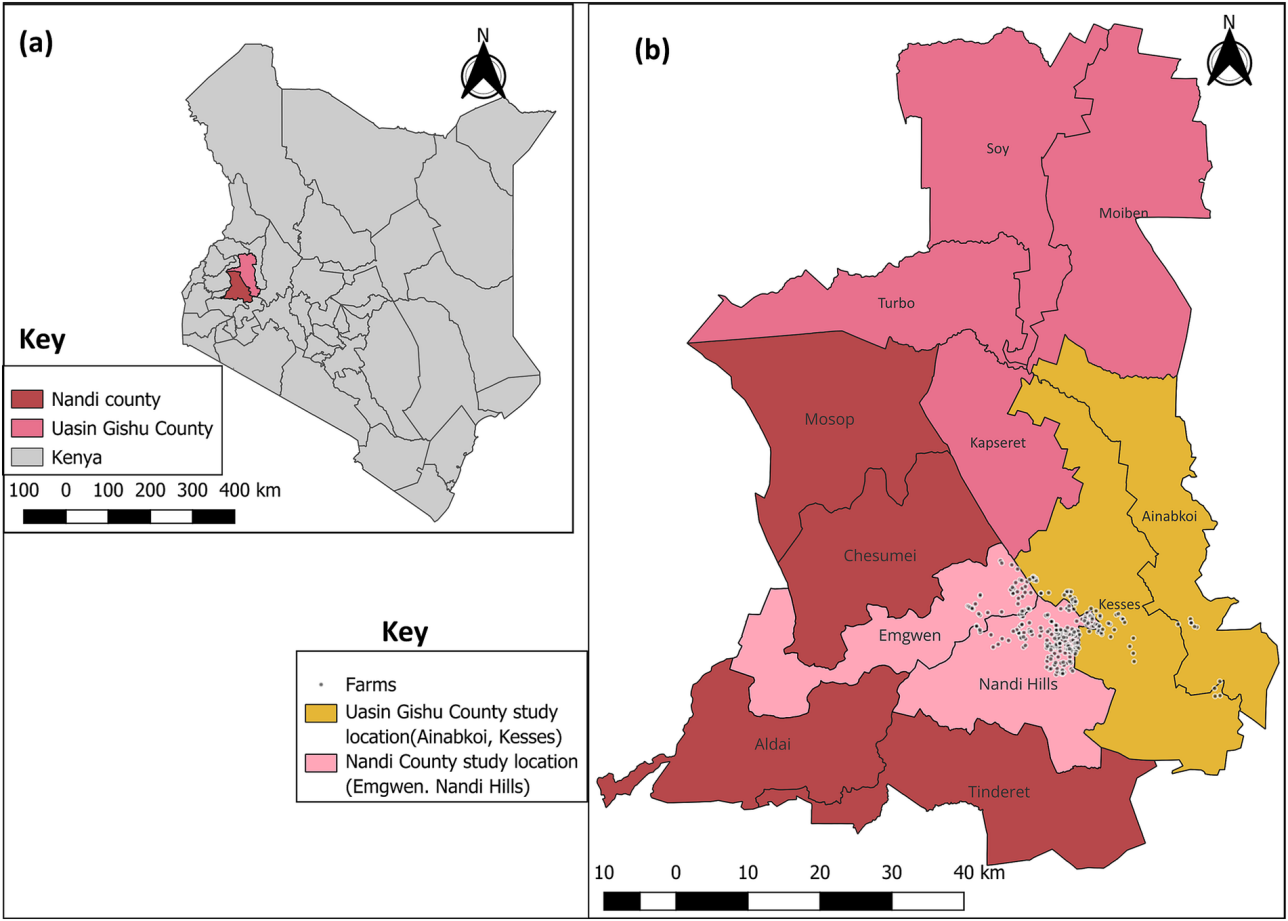
Map of Kenya showing study locations in Nandi and Uasin Gishu County. **(a)** Nandi and Uasin Gishu County and **(b)** Sub-counties sampled (Nandi County: Emgwen, Nandi hills. Uasin Gishu County: Ainabkoi and Kesses). Source. Map was drawn using QGIS version 3.36.0 with the shape files downloaded from Humanitarian Data Exchange database (https://data.humdata.org).

The study was conducted in Nandi county, situated in the North Rift region of Kenya within Agroecological Zone II (upper highlands, lower highlands and upper midlands) (44), covering an area of 2855.8 square kilometers, and including neighboring Kesses and Ainabkoi Sub-counties within the Uasin Gishu County (Figure 1). In this area, the alternating dry and wet seasons, with heavy rains in April (~200 mm) and low rainfall between January and February (~50 mm) (44), provide favorable conditions for dairy farming. Significant investments have been made in local dairy genetic resources and toward controlling the transmission of infectious diseases among this population. Locally, dairy farming accounts for 10% of the national dairy herd (309,038 dairy cattle as of the 2019 National Census) (45), generating an estimated 121.5 million kilograms of milk annually, with a market value of approximately 3.6 billion Kenyan shillings (44). The combination of a favorable climate, widespread grazing-based management, and lack of robust diagnostic methods for GIN make this region particularly suitable for studying nematode co-infections in calves.

### 2.3 Study design and sampling

This study was designed as a cross-sectional study. The sampling frame, collated from the Africa Dairy Genetic Gain (ADGG) database at ILRI,^1^ included 448 farms from the Lessos Farmer Cooperative enrolled in the Centre for Tropical Livestock Genetics and Health (CTLGH) abortion cohort, of which 289 farms with eligible dairy calves were visited for sample collection between September and December 2023. The sample size was calculated as described by Audigé (46), which suggested a minimum of 375 animals.

While the definition of “calf” varies across sources, ranging from age-based cut-offs to criteria focused on the timing of weaning, this study defined calves as animals up to 12-month-old. This broader definition, consistent with the latest FAO guidelines for the World Agricultural Census (47), was adopted to account for local variations in weaning practices, calf growth rates, and their overall maturity, often lagging behind international standards in East Africa (48, 49). Across farms, all calves aged 3 to 12 months were sampled, for a total of 532 calves included in the study. This age range was chosen because it corresponds with a period of increased grazing activity, raising the exposure to pastures potentially contaminated with infective larvae (50). Calves younger than 3 months were not sampled as they primarily depend on nursing, have minimal pasture exposure, and benefit from short-term immunity acquired through colostrum (51).

Fecal samples were collected from each calf using a rectal grab approach to avoid environmental contamination. Samples were placed in an airtight glove, labeled with the calf ID, and transported to the Kapsabet Veterinary Laboratory in cool boxes with ice packs to avoid GIN egg hatching.

### 2.4 Data collection

A structured questionnaire was administered to a consenting adult member of the household (≥18 years) familiar with the herd; all responses were recorded using the Open Data Kit (ODK) system (52). Data collected included several factors at both the animal and farm levels. At the farm level, the type of management system was recorded. Animal-level factors comprised sex, age (estimated in months by the farmer at the time of sampling), heart girth (measured by the animal health assistant), breed (assessed based on phenotype by the animal health assistant), weaning status at the time of sampling, and deworming status within the past 4 weeks, including the type of dewormers used.

### 2.5 Fecal egg count

Fecal Egg Count (FEC) was performed using the modified McMaster technique as described previously by Christie and Jackson (53). Briefly, 1 g of feces was weighed and placed in a sieve bowl, where 15 mL of water was added and mixed. The resulting solution was transferred to a 15 mL centrifuge tube and spun at 1,000 revolutions per minute (rpm) for 2 min. The supernatant was discarded, and the pellet was resuspended in 12 mL of saturated salt solution and centrifuged at 1,000 rpm for 2 min. The supernatant was then transferred to a cuvette and viewed using a microscope at a 10X magnification lens (53). Samples were done in duplicates and the mean FEC was calculated by averaging the results from both runs. The mean eggs per gram (EPG) was then grouped into low intensity (<50), medium intensity (50–200) and high intensity (>200) (20).

### 2.6 First stage larvae (L1) coproculture and isolation

Larval coproculture and isolation was performed as explained by Burgess et al. (54). Briefly, larval coproculture was set on a sample of fresh feces from each calf. This process involved the use of stacked filters with pore sizes of 150 μm, 53 μm, and 30 μm. The filtrate collected from the final 30 μm filter was transferred to a 15 mL tube, and a flotation technique was used to recover the eggs. The recovered eggs were rinsed three times with water, and 3 mL of the suspension was then incubated in 6-well cell culture plates for 48 h.

The incubated solution containing L1 larvae was passed through the filter directly into a 6-well plate and incubated for 2 h at room temperature. The solution was then centrifuged, and the resulting pellet was resuspended in 2 mL of water and stored at −20°C until DNA extraction was performed. After DNA extraction, the L1 was stored in 70% Ethanol (Sigma-Aldrich) and frozen at −80°C for long term storage.

### 2.7 DNA extraction

DNA extraction was performed via the TANBead® nucleic acid extraction system model Maelstrom 9,600 series using the TANBead Nucleic acid Extraction kit (W6T2A46),^2^ which was performed according to the manufacturer’s recommendations and DNA was eluted in 100 μL of elution buffer.

### 2.8 PCR and deep amplicon sequencing of the ITS-2 rDNA locus

PCR and deep amplicon sequencing were performed as described by Avramenko et al. (10). It involved two PCR reactions. The first PCR used NC1 and NC2 primers (Supplementary Table S1). L3 stages of *Haemonchus contortus* and *Teladorsagia circumcincta* (provided by Professor Neil Sargison, Royal (Dick) School of Veterinary Studies, University of Edinburgh), and 9 GIN samples (Roslin Institute, University of Edinburgh), were used as positive controls. Nuclease free water was subsequently used as a negative control to test for contamination. The PCR products were purified using AMPure XP Magnetic Beads (Beckman Coulter, Inc). The second PCR, with limited cycles, was done to add Illumina barcoding indices and P5/P7 tags combinations to each sample (Supplementary Table S2). An aliquot of 10 μL from the second PCR products from each sample were pooled to make a master sequencing library pool and were purified using AMPure XP Magnetic Beads (Beckman Coulter, Inc) according to manufacturer’s recommendation. Finally, 70 μL of the pooled libraries were submitted to Genewiz Genomics, Germany^3^ for Illumina MiSeq sequencing. This was done using a 500-cycle paired-end reagent kit (MiSeq Reagent Kits v2, MS-103-2003) with an addition of 10% PhiX Control v3 (Illumina, FC-110-2003) to generate 250 bp paired-end sequencing reads. Each resequencing step followed Illumina’s standard protocol.

### 2.9 Bioinformatics analysis

The amplicons sequence data was analyzed using the Snakemake pipeline developed for analysis of amplicon sequencing data from MiSeq^4^ which was adapted from Silwamba et al. (55). The raw sequencing reads were trimmed at Phred score of 28 using Sickle (56). The high-quality paired end reads were overlapped and extended using Flash (57) followed by identification of PCR primers and those with exact match with forward and reverse primers were retained. The sequences with 100% identity were clustered to form unique variants present in the data. The variants were then searched against the custom Nematode database and NCBI nr database using BLAST (58). Subsequently, manual curation of the data was performed to confirm the infection. A read number threshold of 500 reads was used to differentiate between true positives and background noise, with read counts below 500 considered negative.

To validate the assay, 36 negative controls were included, two from the first PCR, two from the indexing PCR, and two from DNA extraction for each plate. Of these, 31 showed no reads confirming clear results. The remaining five exhibited very low read numbers, all of which were below the established threshold of 500 reads attached as a Supplementary Table S3. The raw sequencing data was submitted to the European Nucleotide Archive and is available under the project accession number PRJEB85916.

### 2.10 Phylogenetic analysis

The DNA sequences for each species were grouped separately, and duplicate sequences appearing at least twice were merged into a single representative sequence using the FaBox DNA Collapser tool.^5^ Amplicon Sequence Variants (ASV) were aligned using Multiple Sequence Alignment Fast Fourier Transform (MAFFT) with the maximum number of iterations set to 1,000 using the local pairwise alignment algorithm and visualized using SEAVIEW (59, 60). A maximum likelihood phylogenetic tree was then constructed for the ASV of the study samples utilizing IQ-TREE software^6^ with 1,000 bootstraps. TPM2 model was chosen based on the Bayesian Information Criterion (BIC), with a gamma shape parameter of 0.765. The tree was then visualized using the iTOL (Interactive Tree of Life) online tool (61).

### 2.11 Statistical analysis

All data analyses were conducted using R software version 4.3.3^7^ in R Studio (Boston, MA^8^). A descriptive summary of the study population, FEC and heart girth was generated. The *survey* package (62), was used to estimate the design-adjusted true species prevalence, and all estimates were given with a 95% confidence intervals (CI) with adjustment done at the farm level.

Generalized mixed-effects models using the *lme4* package (63) were employed to evaluate risk factors for FEC, heart girth, and co-infections, while accounting for clustering at the farm level. Variables with a *p*-value ≤ 0.2 in the univariable analysis against each outcome were selected for further multivariable analysis. Age was included as a continuous variable in the models.

**Figure 2:**
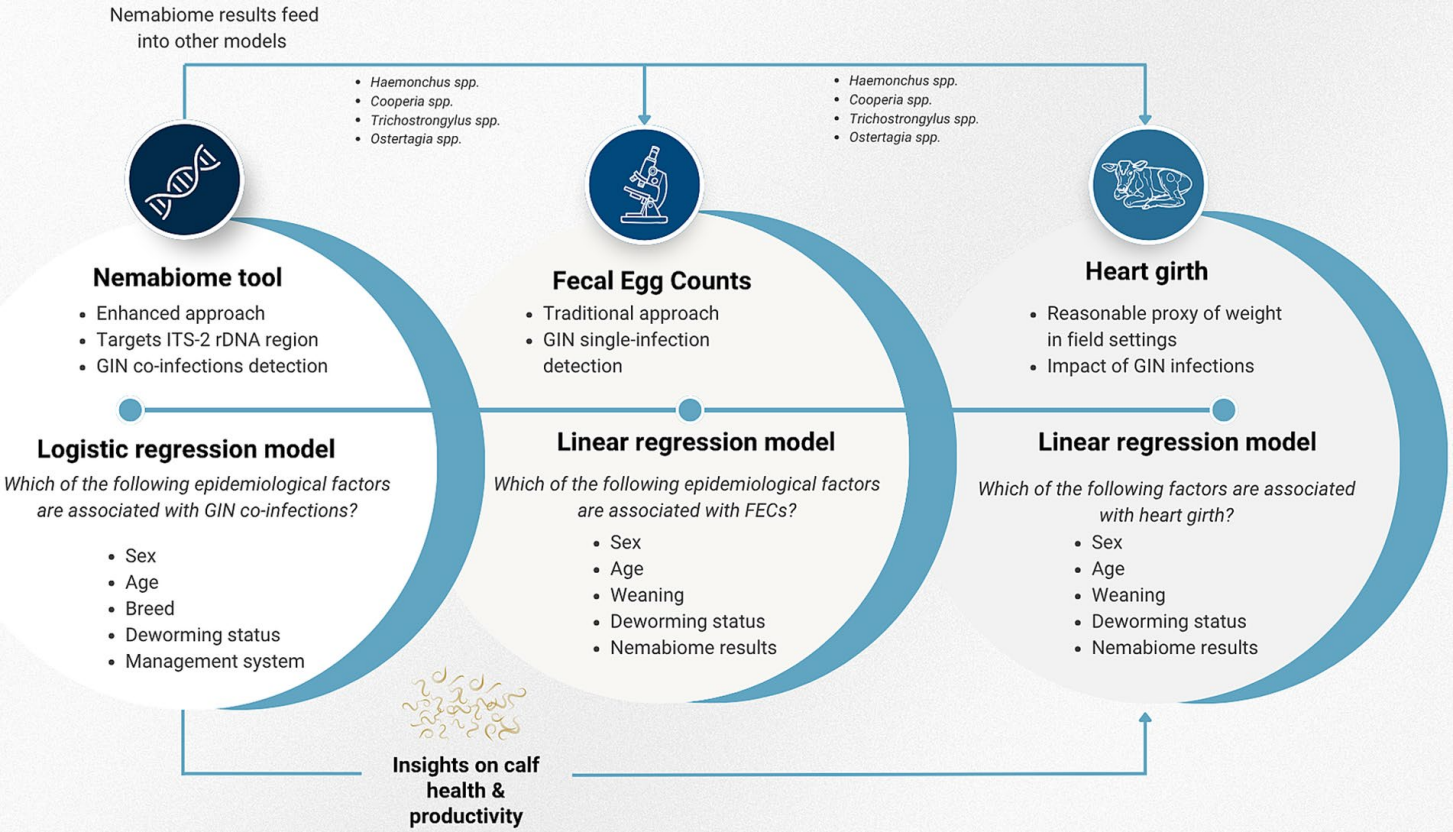
Conceptual framework of the statistical modeling. Three interconnected models were developed. The Nemabiome data feeds into the heart girth model, which assesses health and productivity impacts by evaluating the potential role of specific GIN species on heart girth. Simultaneously, recognizing that FEC is an imperfect diagnostic tool, the Nemabiome results are used as epidemiological factors to explore whether certain species are relevant when interpreting FEC findings.

Three models were developed (Figure 2): (1) a linear regression model for FEC, to identify associated epidemiological factors. FEC data was log-transformed to meet linear model assumptions. This model included six GIN species identified through deep amplicon sequencing to explore species-specific associations with FEC (Supplementary Table S4); (2) a logistic regression model aimed to identify risk factors for nematode co-infections. Animals with single or no infection were categorized as negative, and those with multiple infections were classified as positive (Supplementary Table S5). Finally, (3) a linear regression model for heart girth, a proxy for weight, to assess potential health and productivity impacts of GIN infections, based on the Nemabiome results (Supplementary Table S6). Age, deworming status, and sex were included as key confounding variables in the FEC and co-infection models. Variable selection was conducted using a backward stepwise approach, and Akaike Information Criterion (AIC) was assessed for each model (64).

To measure Alpha diversity among calves, the Shannon-Wiener (H) and Simpson indices (D) were calculated using the vegan package (version 2.6–8) (65). Simpson’s diversity index assesses the evenness of species in a community, its value varies from 0 to 1, with values close to zero suggesting low community diversity. In contrast, the Shannon diversity index considers both richness and evenness within a community, the higher the value, the more diverse the community (66).

## 3 Results

### 3.1 Description of the study population

The study population consisted of 532 dairy calves from 289 farms. Over half of the calves were females (57%) and most were kept under a pasture management system (85.5%). Most calves were young, aged 3–4 months (32.7%) and had been weaned (45.7%) at the time of sampling. Holstein-Friesian crosses (61.3%) were the predominant breed, followed by Ayrshire (36.3%) and Channel Island crosses (2.4%). Most of the calves had been dewormed in the 4 weeks prior to sample collection (63.5%), primarily with benzimidazoles (45.5%) (Supplementary Table S7).

### 3.2 Fecal egg count results

Among the 532 sampled calves, 98.7% (*n* = 525) had at least one egg per gram (EPG), with a mean of 62.0 ± 93.0. Most calves, 64.2% (*n* = 337) had low-intensity FEC (<50 EPG), 28.6% (*n* = 150) had medium intensity (50–200 EPG), and 7.2% (*n* = 38) had high intensity (>200 EPG). The highest mean FEC was observed in male (72.9 ± 104.3 EPG), non-weaned calves (71.0 ± 105.7 EPG), aged 3–4 months (77.5 ± 123.8 EPG), and those that had not been dewormed (67.4 ± 88.5 EPG) (Supplementary Table S8).

Univariable analysis results are provided in Supplementary Table S9. The final multivariable linear regression model for FEC showed that male calves had significantly higher FEC compared to females (estimate = 0.23, 95% CI: 0.02–0.43), while FEC decreased with age (estimate = −0.07, 95% CI −0.11 to −0.03). Calves that were not dewormed had a higher FEC compared to the dewormed calves (estimate = 0.3, 95% CI: 0.04–0.56). *H. placei* (estimate = 0.36, 95% CI: 0.09–0.62) and *C. punctata* (estimate = 0.32, 95% CI: 0.05–0.58) were positively associated with higher FEC (Table 1).

**Table 1:** Generalized mixed effects linear regression model for fecal egg count (FEC) in dairy calves including species associated with FEC.

Variables	Estimates	95% CI
Sex		
Female	Ref	–
Male	0.23	0.02–0.43
Age	−0.07	−0.11 to −0.03
Deworming status		
Yes	Ref	–
No	0.3	0.04–0.56
*Haemonchus contortus*	−0.2	−0.46 to 0.07
*Haemonchus placei*	0.36	0.09–0.62
*Cooperia punctata*	0.32	0.05–0.58

### 3.3 Nemabiome of Kenyan dairy calves

**Figure 3:**
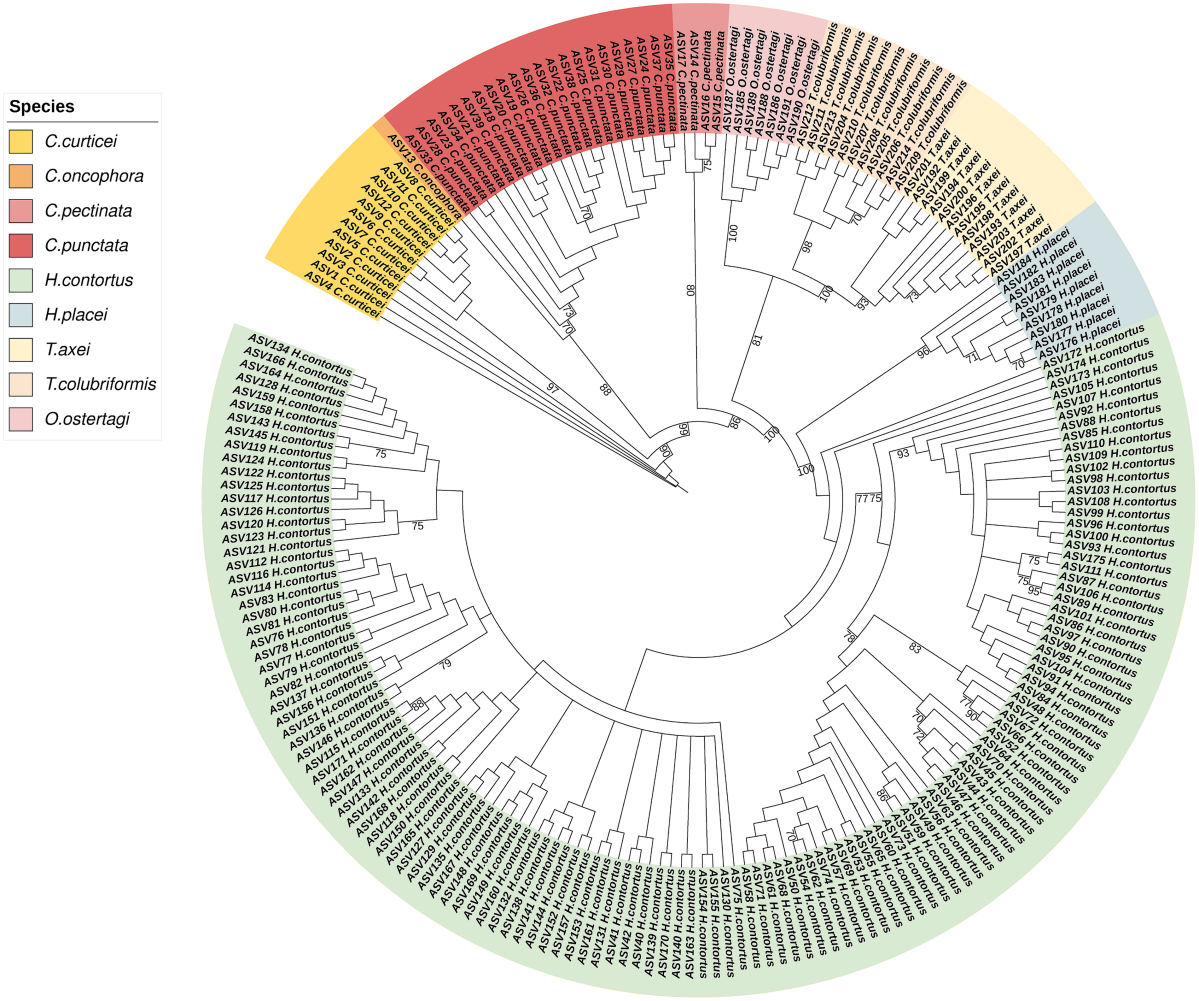
Maximum likelihood phylogenetic tree of ASV from the study samples. ITS-2 rDNA amplicons were sequenced using Illumina MiSeq sequencing. The sequences are differentiated by color, each representing their respective species. The numerical annotations on the branch are bootstrap values and >70 bootstrap value is shown.

A total of 528 samples were submitted for sequencing; out of these, 525 samples were analyzed, three were excluded due to missing linkage to the field IDs. The amplicon sequences had an average read number of 45,181, ranging from 503 to 189,339 reads. Based on sequences analysis we identified 214 Amplicon Sequence Variants (ASV) which were distributed across four genera: *Cooperia, Haemonchus, Trichostrongylus* and *Ostertagia*. Distinct clustering of ASVs was observed, with each major clade corresponding to a distinct trichostrongyloid GIN species (Figure 3). *Haemonchus contortus* was the most diverse species with more ASV, which reflect a high level of intraspecific variation. This was followed by *Cooperia punctata, Cooperia curticei, Trichostrongylus axei, Trichostrongylus colubriformis, Haemonchus placei* and *Ostertagia ostertagi* which constituted smaller but distinct clusters, likely indicating lower sequences diversity within these species. *Cooperia pectinata* and *Cooperia oncophora* exhibited the lowest ASV counts with 4 and 1 ASVs, respectively.

The prevalence of GIN from deep amplicon sequencing results was 49.9% (95% CI: 44.8–55.0; *n* = 262). Among the species identified, *C. punctata* was the most prevalent at 27.8% (95% CI: 23.5–32.5), followed by *H. placei* 26.3% (95% CI: 22.2–30.8), *H. contortus* 23.6% (95% CI: 19.6–28.1), *T. axei* 16.4% (95% CI: 13.1–20.3), *O. ostertagi* 14.5% (95% CI: 11.5–18.1), *T. colubriformis* 8.0% (95% CI: 5.8–10.9), *C. curticei* 2.9% (95% CI: 1.7–4.8), *C. pectinata* 2.5% (95% CI: 1.3–4.7), and *C. oncophora* 0.2% (95% CI: 0.0–1.3). The proportion of unique nematode species and read abundance per animal is provided in Supplementary Figure S1.

**Figure 4:**
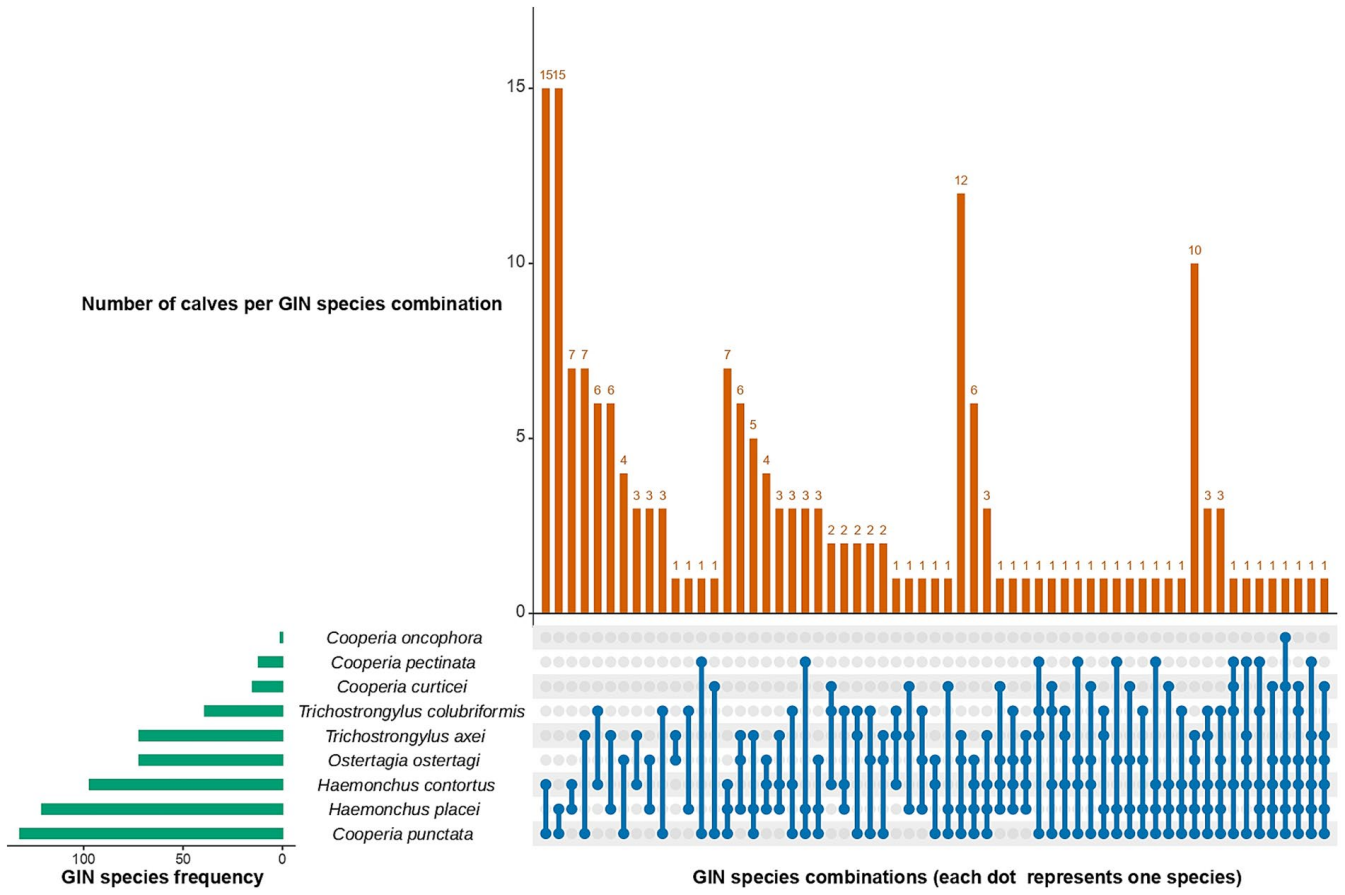
UpSet plot showing co-infection patterns for gastrointestinal nematodes (GIN) in dairy calves. The horizontal bars (left) represent the total number of samples positive for each species, while the vertical bars (top) indicate the number of calves with specific species combinations. Dots connected by lines at the bottom indicate species combinations.

Nemabiome results showed that, 30.5% of calves (*n* = 80) had single infections, while 69.5% (*n* = 182) were infected with two or more GIN species. Among these, two-species combinations were the most common (*n* = 73), followed by three-species combinations (*n* = 49), four-species combinations (*n* = 36), five-species combinations (*n* = 20), and finally, six-species combination (*n* = 4). For two-species infections, *C. punctata* frequently paired with *H. contortus* and *H. placei* (*n* = 15) whereas *C. punctata*, *H. contortus*, and *H. placei* were the most common three-species infections (*n* = 7). In four-species infections, the dominant group consisted of *C. punctata*, *H. placei*, *O. ostertagi*, and *T. axei* (*n* = 12). For five-species infections, *C. punctata*, *H. contortus*, *H. placei*, *O. ostertagi*, and *T. axei* dominated (*n* = 10) (Figure 4).

Univariable analysis results for the co-infection model are provided in Supplementary Table S10. The final multivariable logistic regression model for co-infections showed that calves managed under a pasture system had twice the odds (OR = 2.52, 95% CI: 1.17–5.40) of experiencing mixed infections compared to those in zero-grazing systems. Age, deworming status, management system and sex were not significantly associated with co-infections (Table 2).

**Table 2:** Generalized mixed effects logistic regression model for co-infections in dairy calves.

Variables	Odds ratio (OR)	95% CI
Sex		
Female	Ref	–
Male	1.0	0.64–1.56
Age	0.99	0.91–1.07
Deworming status		
Dewormed	Ref	–
Not dewormed	0.60	0.36–1.01
Management system		
Zero Grazing	Ref	–
Pasture	2.52	1.17–5.40

*For the management system, pasture includes also cut-and-carry.

### 3.4 Effect of GIN species and other factors on heart girth in dairy calves

The mean heart girth for all calves was 99.2 ± 14 cm (Supplementary Table S11). Univariable analysis results for the heart girth model are provided in Supplementary Table S12. The final multivariable linear regression model for heart girth showed that male calves had a significantly smaller girth compared to females (estimate = −2.61, 95% CI: −4.64 to −0.58). Age was positively associated with heart girth (estimate = 2.47, 95% CI: 2.06–2.89). Infections with *O. ostertagi* (estimate = −4.43, 95% CI: −7.65 to −1.20) and *T. colubriformis* (estimate = −3.96, 95% CI: −7.68 to −0.24) were associated with a decrease in girth (Table 3).

**Table 3:** Generalized mixed effects linear regression model for heart girth in dairy calves.

Variables	Estimates	95% CI
Sex		
Female	Ref	–
Male	−2.61	−4.64 to −0.58
Age	2.47	2.06–2.89
Weaning		
Not weaned	Ref	–
Weaned	1.40	−1.03 to 3.83
Deworming status		
Dewormed	Ref	–
Not dewormed	0.76	−1.58 to 3.09
*Haemonchus placei*	2.43	−0.17 to 5.02
*Ostertagia ostertagi*	−4.43	−7.65 to −1.20
*Trichostrongylus colubriformis*	−3.96	−7.68 to −0.24

### 3.5 Alpha diversity indices

**Figure 5:**
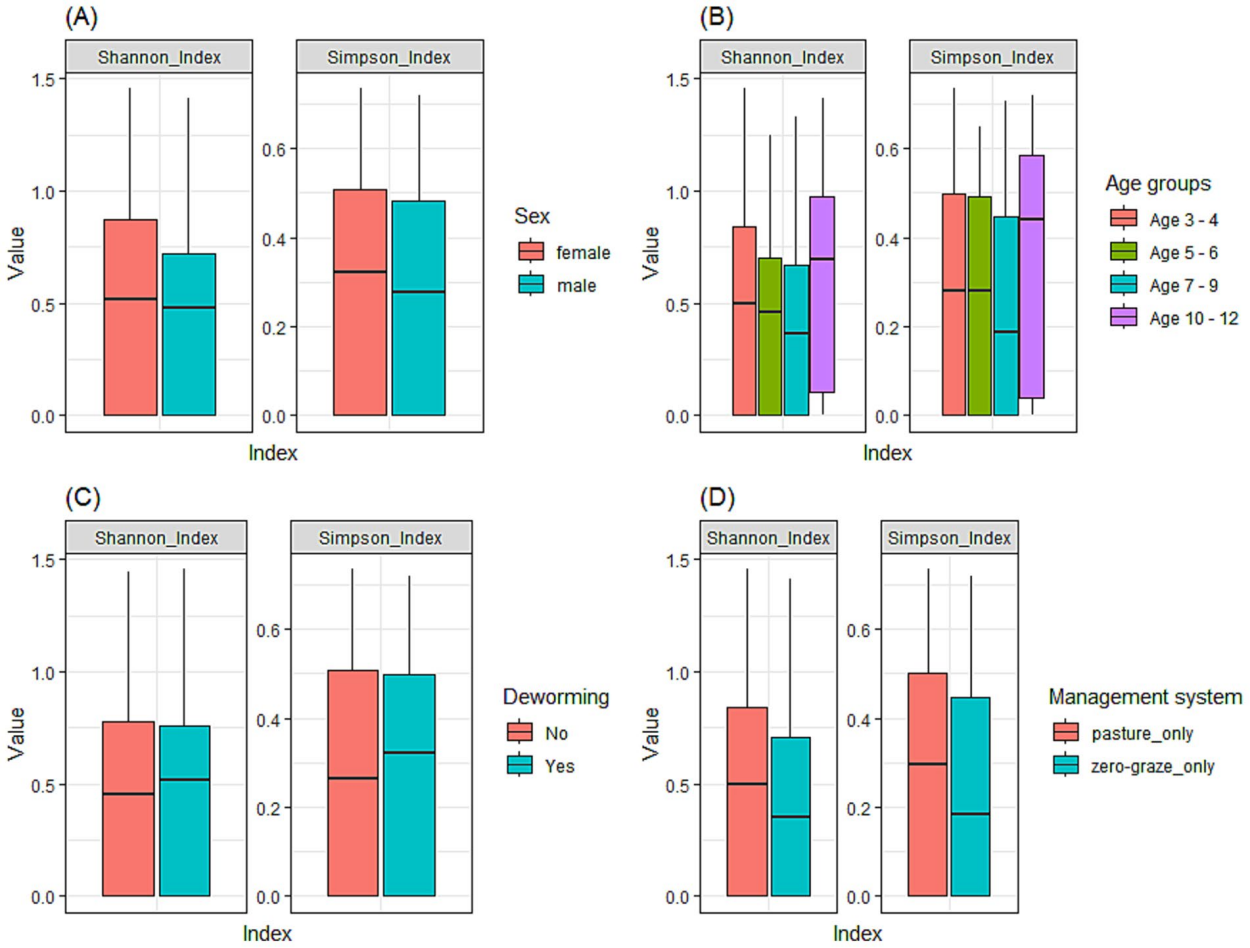
Alpha diversity of GIN communities in dairy calves using the Shannon and Simpson indices to compare diversity across various factors. **(A)** sex; **(B)** age groups (3–4, 5–6, 7–9, and 10–12 months); **(C)** deworming status; **(D)** management system.

The Shannon Index ranged from 0 to 1.5, with a mean of 0.49 ± 0.43. In contrast, the Simpson Index ranged from 0 to 0.7 and a mean of 0.3 ± 0.25. Figure 5 illustrates Shannon and Simpson index values in relation to sex (Figure 5A), age (Figure 5B), deworming status (Figure 5C) and management system (Figure 5D). While the results were not statistically significant (*p* > 0.05) for any of the variables assessed, notable patterns were observed across several variables. Specifically, the 10–12 months age group presented slightly higher Shannon index values compared to the younger calves (3–4 months and 5–6 months), suggesting a tendency to increased GIN diversity with age. Female animals exhibited marginally greater GIN diversity than males. A modest increase in diversity was also observed in calves managed under pasture-grazing systems compared to those under zero-grazing management. Interestingly, calves that had been dewormed showed slightly higher GIN diversity than those that had not been dewormed.

## 4 Discussion

Microscopy-based diagnostic tests, remain essential for detecting GIN infections and estimating infection intensity, particularly for everyday diagnostic purposes. However, high-throughput molecular methods such as the Nemabiome allow for a robust and precise identification of individual GIN species and capture the complexity of co-infections, often undetected by conventional methods like FEC (10).

The mean FEC in this study was higher than the FEC reported in smallholder dairy farms in Nakuru and Nyeri Counties (48.0 EPG) (7), but lower than a study conducted in smallholder farms in Kirinyaga County (88.0 EPG) (37) in Kenya. In general, while the prevalence of FEC (i.e., the proportion of calves with at least one EPG) was high, the intensity of infection was generally low, with most of the calves in our study having less than 200 EPG. The different findings across FEC-based studies could be due to variations on the study design, test method, analyst experience, study population, host genetics, management system, and geoclimatic conditions (20, 67, 68).

Earlier studies have demonstrated that age significantly influences FEC (35, 69, 70), with young calves representing the highest-risk subpopulation for GIN infection (67), consistent with the findings of this study. Lower immunity among younger calves increases their susceptibility to various infections. Primary exposure to GIN species typically triggers a strong protective immune response, which significantly reduces the risk of GIN reinfection over time and contributes to reduced parasite transmission within pasture-based dairy herds (67, 69). In contrast, literature reports mixed findings regarding FEC differences between male and female calves. Some studies indicate males have a higher GIN burden as detected through FEC (36, 71), while others report no significant difference in infection rates between male and female, such as in pasture-kept indigenous cattle (39). In this study, the observed sex-related difference in FEC may be partially attributed to management practices in dairy farms, where male calves are usually weaned earlier and may experience undernutrition, leading to increased susceptibility to infections and stunted growth compared to their female counterparts (48, 49, 72).

Microscopic examination has previously identified multiple GIN species in Kenyan livestock (7, 37, 43). Using these traditional methods, the most common species belonged to *Haemonchus*, *Cooperia*, *Trichostrongylus*, *Oesophagostomum*, and *Nematodirus* genera. In contrast, through the nemabiome approach, this study identified nine different GIN species isolated from the L1, four *Cooperia* species (*C. curticei*, *C. oncophora*, *C. pectinata*, and *C. punctata*), two *Haemonchus* species (*H. contortus* and *H. placei*), two *Trichostrongylus* species (*T. axei* and *T. colubriformis*) and one *Ostertagia* species (*O. ostertagi*). Unlike previous studies in Kenya (7, 37, 42, 43, 73, 74), our study detected *O. ostertag*i, which had not been reported earlier. Prevalent in temperate regions*, O. ostertagi* has been documented in tropical cattle in Nigeria (36), South Africa (38) and Tanzania (40). We did not detect any *Oesophagostomum* species such as *Oesophagostomum radiatum,* which has been documented in past studies (7, 37, 43). Discrepancies across studies may be attributed to dissemination of parasites through animal movements (75), differential host susceptibility linked to genetics (67), pasture management strategies, anthelmintic protocols, and ecological factors such as climate change, that affect the environmental suitability for GIN survival across ecological niches (76). It also reflects the diagnostic performance of the approach used, with the nemabiome being a more reliable, high-resolution tool for species identification compared to traditional morphological and coproculture methods (10).

*Haemonchus* species are recognized for their pathogenicity in livestock, causing lesions in the abomasum and severe anaemia due to its blood-feeding behavior (77). Among these, *H. placei* and *H. contortus* emerge as the most pathogenic species (78). *H. placei* is typically found in cattle, although mixed infections with *H. contortus* can also occur (79). While *H. contortus* is regarded as the most significant internal parasite of sheep and goats, it can also infect cattle (80), with calves being highly susceptible (77). The co-rearing of cattle with small ruminants, a common practice in dairy farms within the study region, may have played a role in this local transmission dynamic.

*T. colubriformis* and *T. axei* species are known to infect both humans and livestock (81). In humans, adult worms of *Trichostrongylus* species inhabit the mucosa of the small intestine and in large numbers, can induce physical trauma leading to mucosal shedding, and hemorrhages in severe cases (82). In contrast, in cattle it lives in the abomasum, typically part of a mixed helminth infection, with clinical symptoms that include diarrhea, anorexia, progressive weight loss, and general weakness (83). Studies in Iran, Australia, and Thailand have reported infections in humans living near animals (84–86). In Kenya, *T. colubriformis* has been recorded in baboons in the Amboseli region, suggesting its presence in various hosts (87). Since GIN are soil-transmitted, they can contaminate the environment, leading to increased exposure among the human population (88). Using cow or sheep manure as fertilizer can further contribute to the spread of infection in farming communities, elevating the risk of zoonotic transmission (89). This can result in a higher incidence of infections, particularly among rural communities with poor hygiene standards and limited access to health services (81). The occurrence of *Trichostrongylus species* in livestock and wildlife raises public health concerns in Kenya, where little information is available about the burden of nematode infections among farming communities.

FEC findings are influenced by the burden of infection, species composition and fecundity of GIN, and individual differences in the immune-response (90), as well as the time of sampling (91). For instance, *Haemonchus* and *Cooperia* are highly fecund species compared to *Ostertagia* (20). Despite producing fewer eggs, *Ostertagia* is still highly pathogenic highlighting a key limitation of the FEC as a diagnostic method. In cases of co-infection, FEC results may be further misleading because different nematode eggs can appear similar under a microscope. If the person analysing the sample lacks expertise, they may misidentify the species, potentially leading to inaccurate identification and impacting the therapeutic plan.

Morphological measurements, such as heart girth, are valuable proxies for estimating livestock live weight, particularly in field settings where weighing scales are impractical (92–94). Heart girth has been validated in various contexts, including crossbred dairy cattle in Sub-Saharan Africa, and is consistently presented as an accurate tool across age and breed groups (93, 94). However, animal genetics, husbandry practices, and nutrition around weaning can influence growth performance. Along with individual immune responses and GIN parasitic loads, these factors collectively impact the type and severity of clinical signs and girth measurements (72, 95, 96).

Heavy or chronic parasitism can impair growth and weight gain by exacerbating the physiological stress associated with GIN infestation (48), which may partly explain the observed reduction in heart girth among male calves. For instance, *O. ostertagi* infection in cattle is often associated with chronic diarrhea and weight loss, especially during the first grazing season (97, 98). Similarly, infection with *T. colubriformis* is known to impair feed efficiency and nutrient absorption, leading to growth retardation, even in cases of subclinical GIN parasitism (99). Differences in management practices may also play a role. In dairy-oriented regions, farmers tend to provide more care to female calves due to their value as replacement stock and their importance to the dairy enterprise (96). These findings underscore the importance of effective, integrated GIN control in the farm to interrupt transmission cycles of GIN shared by calves, adult animals, and other hosts.

Mixed parasitic infections are common in pasture-managed cattle (36, 100, 101). Favorable climatic conditions provides a conducive environment for GIN to mature (102), leading to pasture contamination and increasing the likelihood of calves being exposed to infective larvae. Co-infection with multiple parasites can influence both the spread and severity of diseases; parasite interactions may alter the immune response of the host, making them more susceptible to other infections (103). For instance, Thumbi et al. (104) highlighted that the risk of death from the infection by *Theileria parva* (causative agent of East Coast fever) significantly increased with a high helminth burden measured as strongyle EPG. Nematode infections in buffalos have also been linked to increased vulnerability to diseases like bovine tuberculosis (bTB), associated with a reduction in the Th1 immune response (105).

The alpha diversity indices revealed no statistically significant differences in GIN species among calves in the study region. This suggests that the overall diversity of GIN in dairy calves remains relatively stable, regardless of features such as sex or age. Increased GIN exposure and continuous reinfection cycles occur with prolonged grazing periods, after maternal immunity wanes, allowing for colonization by multiple parasite species (67). The slightly higher diversity observed in dewormed animals is a counterintuitive finding, potentially arising from combination of recall bias among smallholders regarding their anthelmintic schedules, or alternatively indicating reinfection from contaminated pasture, where deworming without proper pasture management facilitates GIN host repopulation (106). This finding also highlights the advantages of using the Nemabiome tool as the next-generation approach to GIN characterization compared to traditional methods. Further research is needed to better understand the observed patterns, particularly integrating emerging anthelmintic resistance with ecological and husbandry factors.

Our study offers valuable insights into the prevalence, predominant GIN species, and co-infections affecting calves in one of the most important dairy regions in Kenya. The increasing use of the Nemabiome tool to monitor GIN diversity and anthelmintic resistance in high-income countries (HICs) (3, 10, 16, 30, 31, 107, 108), highlights emerging opportunities for its application in tropical regions, where the burden of infection is typically higher. This aligns with the growing need for sustainable and safe strategies to manage GIN, particularly considering the cost of anthelmintics, the increase in drug resistance, and consumer concerns about animal welfare and organic animal products. Although the Nemabiome technique remains relatively costly for widespread implementation in resource-limited settings, it becomes more cost-effective with larger sample sizes and serves as a powerful tool for evidence-based practice (10).

Future research should focus on assessing the GIN burden in calves under 3 months of age, who were not included in this study, and conduct longitudinal surveys to capture seasonal variations and long-term trends related to the impact of GIN infections on calf and heifer health, welfare, and productivity.

## 5 Conclusion

This study is the first to the assess the burden and diversity of gastrointestinal nematodes in dairy calves in Africa using a combination of fecal egg count (FEC) analysis and the Nemabiome assay using L1. In the study region, age, deworming, and management practices were found to influence the nematode burden, which in turn had an impact on heart girth, a proxy for weight. Significant associations between infections with *O. ostertagi* and *T. colubriformis* and reduced heart girth were identified, underscoring the clinical significance of the findings. Due to the complex epidemiology of GIN in calves in the region, characterized by frequent co-infections, this study also highlights the need to evaluate the efficacy of commonly used anthelminthics to support the design of a sustainable GIN control strategy.

## Data Availability

The datasets presented in this study can be found in online repositories. The names of the repository/repositories and accession number(s) can be found at: https://www.ebi.ac.uk/ena, PRJEB85916 and the animal metadata and test results are available at https://doi.org/10.3389/fvets.2025.1588350.

## Footnotes

^1^ https://portal.adgg.ilri.org/

^2^ https://www.tanbead.com/en

^3^ https://www.genewiz.com

^4^ https://github.com/deepalivasoya/MiAmp

^5^ https://users-birc.au.dk/palle/php/fabox/dnacollapser.php

^6^ http://www.iqtree.org/

^7^ https://www.R-project.org/

^8^ http://www.rstudio.com/
