# Supplementary material for: Species diversity and risk factors of gastrointestinal nematodes in smallholder dairy calves in Kenya
Source: Front Vet Sci. 2025 Aug 12;12:1588350. doi: 10.3389/fvets.2025.1588350 (PMC12379043; doi:10.3389/fvets.2025.1588350)
Supplement: Supplementary file 1 [file Table_1.docx]

Supplementary Material

**Supplementary Figures and Tables**

**1.1 Supplementary Figures**

**Supplementary Figure S1:** Plot showing the relative proportions of ITS-2rDNA sequence reads mapped to parasitic gastrointestinal nematodes among dairy calves in Nandi County (September-December 2023). Each bar represents a single animal as indicated by their respective animal ID, with the height of the bar corresponding to the abundance of each GIN species and the color distinguishing the identified species. Many of the samples show signs of mixed infections, as shown by the different colors in each bar.

**1.2. Supplementary Tables**

***Supplementary Table S1:*** *Primer sequences for Illumina Miseq Library preparation for nemabiome approach. NC1, NC2 and adapters (ADP) primers are bolded, N are underlined.*

| **Primer name** | **Primer sequence (5`-3`)** |
| --- | --- |
| NC1ADP | TCGTCGGCAGCGTCAGATGTGTATAAGAGACAG**ACGTCTGGTTCAGGGTTGTT** |
| NC1ADP1N | TCGTCGGCAGCGTCAGATGTGTATAAGAGACAGN**ACGTCTGGTTCAGGGTTGTT** |
| NC1ADP2N | TCGTCGGCAGCGTCAGATGTGTATAAGAGACAGNN**ACGTCTGGTTCAGGGTTGTT** |
| NC1ADP3N | TCGTCGGCAGCGTCAGATGTGTATAAGAGACAGNNN**ACGTCTGGTTCAGGGTTGTT** |
| NC2ADP | GTCTCGTGGGCTCGGAGATGTGTATAAGAGACAG**TTAGTTTCTTTTCCTCCGCT** |
| NC2ADP1N | GTCTCGTGGGCTCGGAGATGTGTATAAGAGACAGN**TTAGTTTCTTTTCCTCCGCT** |
| NC2ADP2N | GTCTCGTGGGCTCGGAGATGTGTATAAGAGACAGNN**TTAGTTTCTTTTCCTCCGCT** |
| NC2ADP3N | GTCTCGTGGGCTCGGAGATGTGTATAAGAGACAGNNN**TTAGTTTCTTTTCCTCCGCT** |

***Supplementary Table S2:*** *Sequences for forward and reverse barcoded primers (Nextera XT*

*Index Kit v2). Illumina indexing tags are bolded.*

| Primer | Sequence, 5’-3’ |
| --- | --- |
| S502_i5 | AATGATACGGCGACCACCGAGATCTACAC**CTCTCTAT**TCGTCGGCAGCGTC |
| S503_i5 | AATGATACGGCGACCACCGAGATCTACAC**TATCCTCT**TCGTCGGCAGCGTC |
| S505_i5 | AATGATACGGCGACCACCGAGATCTACAC**GTAAGGAG**TCGTCGGCAGCGTC |
| S506_i5 | AATGATACGGCGACCACCGAGATCTACAC**ACTGCATA**TCGTCGGCAGCGTC |
| S507_i5 | AATGATACGGCGACCACCGAGATCTACAC**AAGGAGTA**TCGTCGGCAGCGTC |
| S508_i5 | AATGATACGGCGACCACCGAGATCTACAC**CTAAGCCT**TCGTCGGCAGCGTC |
| S510_i5 | AATGATACGGCGACCACCGAGATCTACAC**CGTCTAAT**TCGTCGGCAGCGTC |
| S511_i5 | AATGATACGGCGACCACCGAGATCTACAC**TCTCTCCG**TCGTCGGCAGCGTC |
| S513_i5 | AATGATACGGCGACCACCGAGATCTACAC**TCGACTAG**TCGTCGGCAGCGTC |
| S515_i5 | AATGATACGGCGACCACCGAGATCTACAC**TTCTAGCT**TCGTCGGCAGCGTC |
| S516_i5 | AATGATACGGCGACCACCGAGATCTACAC**CCTAGAGT**TCGTCGGCAGCGTC |
| S517_i5 | AATGATACGGCGACCACCGAGATCTACAC**GCGTAAGA**TCGTCGGCAGCGTC |
| S518_i5 | AATGATACGGCGACCACCGAGATCTACAC**CTATTAAG**TCGTCGGCAGCGTC |
| S520_i5 | AATGATACGGCGACCACCGAGATCTACAC**AAGGCTAT**TCGTCGGCAGCGTC |
| S521_i5 | AATGATACGGCGACCACCGAGATCTACAC**GAGCCTTA**TCGTCGGCAGCGTC |
| S522_i5 | AATGATACGGCGACCACCGAGATCTACAC**TTATGCGA**TCGTCGGCAGCGTC |
| N701_i7 | CAAGCAGAAGACGGCATACGAGAT**TCGCCTTA**GTCTCGTGGGCTCGG |
| N702_i7 | CAAGCAGAAGACGGCATACGAGAT**CTAGTACG**GTCTCGTGGGCTCGG |
| N703_i7 | CAAGCAGAAGACGGCATACGAGAT**TTCTGCCT**GTCTCGTGGGCTCGG |
| N704_i7 | CAAGCAGAAGACGGCATACGAGAT**GCTCAGGA**GTCTCGTGGGCTCGG |
| N705_i7 | CAAGCAGAAGACGGCATACGAGAT**AGGAGTCC**GTCTCGTGGGCTCGG |
| N706_i7 | CAAGCAGAAGACGGCATACGAGAT**CATGCCTA**GTCTCGTGGGCTCGG |
| N707_i7 | CAAGCAGAAGACGGCATACGAGAT**GTAGAGAG**GTCTCGTGGGCTCGG |
| N710_i7 | CAAGCAGAAGACGGCATACGAGAT**CAGCCTCG**GTCTCGTGGGCTCGG |
| N711_i7 | CAAGCAGAAGACGGCATACGAGAT**TGCCTCTT**GTCTCGTGGGCTCGG |
| N712_i7 | CAAGCAGAAGACGGCATACGAGAT**TCCTCTAC**GTCTCGTGGGCTCGG |
| N714_i7 | CAAGCAGAAGACGGCATACGAGAT**TCATGAGC**GTCTCGTGGGCTCGG |
| N715_i7 | CAAGCAGAAGACGGCATACGAGAT**CCTGAGAT**GTCTCGTGGGCTCGG |

***Supplementary Table S3:*** *Sequenced and processed reads numbers of Control samples through the metabarcoding bioinformatics pipeline. Positive control samples are highlighted in bold. Hcon: H. contortus, Tcir: T. circumcincta. Pcr: Control negatives from PCR, DNA: Control negatives from DNA extraction.*

| Control Samples ID | Total sequencing reads | Reads with PCR primers | *Cooperia curticei* | *Cooperia oncophora* | *Cooperia pectinata* | *Cooperia punctata* | *Haemonchus contortus* | *Haemonchus placei* | *Ostertagia ostertagi* | *Trichostrongylus axei* | *Teladorsagia circumcincta* | *Trichostrongylus colubriformis* |
| --- | --- | --- | --- | --- | --- | --- | --- | --- | --- | --- | --- | --- |
| **ctrl-pos1-plt1-1890D** | 57914 | 50958 | 0 | 0 | 0 | 0 | 0 | 0 | 0 | 36463 | 0 | 0 |
| **ctrl-pos2-plt1-1894D** | 3304 | 686 | 0 | 0 | 0 | 0 | 0 | 0 | 0 | 0 | 0 | 0 |
| ctrl-neg1-pcr2-plt1 | 10 | 0 | 0 | 0 | 0 | 0 | 0 | 0 | 0 | 0 | 0 | 0 |
| ctrl-neg2-pcr2-plt1 | 11 | 0 | 0 | 0 | 0 | 0 | 0 | 0 | 0 | 0 | 0 | 0 |
| ctrl-neg1-pcr1-plt1 | 151 | 23 | 0 | 0 | 0 | 0 | 0 | 0 | 0 | 0 | 0 | 0 |
| ctrl-neg2-pcr1-plt1 | 19 | 6 | 0 | 0 | 0 | 0 | 0 | 0 | 0 | 0 | 0 | 0 |
| ctrl-neg1-dna-plt1 | 689 | 270 | 0 | 0 | 0 | 0 | 0 | 0 | 0 | 0 | 0 | 0 |
| ctrl-neg2-dna-plt1 | 265 | 82 | 0 | 0 | 0 | 0 | 0 | 0 | 0 | 0 | 0 | 0 |
| **ctrl-pos1-plt2-Hcon** | 247110 | 227983 | 0 | 0 | 0 | 0 | 124071 | 0 | 0 | 0 | 0 | 0 |
| **ctrl-pos2-plt2-Tcir** | 289424 | 265743 | 0 | 0 | 0 | 0 | 0 | 0 | 0 | 0 | 88800 | 0 |
| ctrl-neg1-pcr2-plt2 | 79 | 26 | 0 | 0 | 0 | 0 | 0 | 0 | 0 | 0 | 0 | 0 |
| ctrl-neg2-pcr2-plt2 | 121 | 37 | 0 | 0 | 0 | 0 | 0 | 0 | 0 | 0 | 0 | 0 |
| ctrl-neg1-pcr1-plt2 | 1005 | 144 | 0 | 0 | 0 | 0 | 0 | 0 | 0 | 0 | 0 | 0 |
| ctrl-neg2-pcr1-plt2 | 272 | 104 | 0 | 0 | 0 | 0 | 0 | 0 | 0 | 0 | 0 | 0 |
| ctrl-neg1-dna-plt2 | 1801 | 347 | 0 | 0 | 0 | 0 | 0 | 0 | 0 | 0 | 0 | 0 |
| ctrl-neg2-dna-plt2 | 190 | 79 | 0 | 0 | 0 | 0 | 0 | 0 | 0 | 0 | 0 | 0 |
| **ctrl-pos1-plt3-1895A-L3** | 44145 | 41126 | 0 | 0 | 14624 | 0 | 0 | 0 | 0 | 0 | 0 | 0 |
| **ctrl-pos2-plt3-1897B-L3** | 11882 | 10981 | 0 | 0 | 1116 | 0 | 0 | 0 | 0 | 3781 | 0 | 0 |
| ctrl-neg1-pcr2-plt3 | 10 | 2 | 0 | 0 | 0 | 0 | 0 | 0 | 0 | 0 | 0 | 0 |
| ctrl-neg2-pcr2-plt3 | 26 | 8 | 0 | 0 | 0 | 0 | 0 | 0 | 0 | 0 | 0 | 0 |
| ctrl-neg1-pcr1-plt3 | 258 | 65 | 0 | 0 | 0 | 0 | 0 | 0 | 0 | 0 | 0 | 0 |
| ctrl-neg2-pcr1-plt3 | 1514 | 798 | 0 | 0 | 0 | 0 | 0 | 0 | 0 | 0 | 0 | 0 |
| ctrl-neg1-dna-plt3 | 8528 | 1996 | 0 | 0 | 0 | 0 | 0 | 0 | 0 | 0 | 0 | 0 |
| ctrl-neg2-dna-plt3 | 243 | 111 | 0 | 0 | 0 | 0 | 0 | 0 | 0 | 0 | 0 | 0 |
| **ctrl-pos1-plt4-1890D-L3** | 22294 | 18643 | 0 | 0 | 0 | 0 | 0 | 0 | 0 | 11583 | 0 | 0 |
| **ctrl-pos2-plt4-1894D-L3** | 29936 | 27959 | 0 | 0 | 7305 | 9070 | 0 | 0 | 0 | 0 | 0 | 0 |
| ctrl-neg1-pcr2-plt4 | 3 | 1 | 0 | 0 | 0 | 0 | 0 | 0 | 0 | 0 | 0 | 0 |
| ctrl-neg2-pcr2-plt4 | 2 | 0 | 0 | 0 | 0 | 0 | 0 | 0 | 0 | 0 | 0 | 0 |
| ctrl-neg1-pcr1-plt4 | 31 | 17 | 0 | 0 | 0 | 0 | 0 | 0 | 0 | 0 | 0 | 0 |
| ctrl-neg2-pcr1-plt4 | 4033 | 695 | 0 | 0 | 0 | 0 | 0 | 0 | 0 | 0 | 0 | 0 |
| ctrl-neg1-dna-plt4 | 8 | 1 | 0 | 0 | 0 | 0 | 0 | 0 | 0 | 0 | 0 | 0 |
| ctrl-neg2-dna-plt4 | 3930 | 1358 | 0 | 0 | 0 | 0 | 0 | 0 | 0 | 0 | 0 | 0 |
| **ctrl-pos1-plt5-1895A-L3** | 52 | 18 | 0 | 0 | 0 | 0 | 0 | 0 | 0 | 0 | 0 | 0 |
| **ctrl-pos2-plt5-1897B-L3** | 4434 | 4028 | 0 | 0 | 2349 | 0 | 0 | 0 | 0 | 0 | 0 | 0 |
| ctrl-neg1-pcr2-plt5 | 21 | 9 | 0 | 0 | 0 | 0 | 0 | 0 | 0 | 0 | 0 | 0 |
| ctrl-neg2-pcr2-plt5 | 13 | 5 | 0 | 0 | 0 | 0 | 0 | 0 | 0 | 0 | 0 | 0 |
| ctrl-neg1-pcr1-plt5 | 74 | 24 | 0 | 0 | 0 | 0 | 0 | 0 | 0 | 0 | 0 | 0 |
| ctrl-neg2-pcr1-plt5 | 289 | 204 | 0 | 0 | 0 | 0 |  | 156 | 0 | 0 | 0 | 0 |
| ctrl-neg1-dna-plt5 | 632 | 473 | 0 | 0 | 0 | 0 | 164 | 0 | 0 | 0 | 0 | 0 |
| ctrl-neg2-dna-plt5 | 25 | 9 | 0 | 0 | 0 | 0 | 0 | 0 | 0 | 0 | 0 | 0 |
| **ctrl-pos1-plt6-1895A-L3** | 176087 | 160558 | 0 | 0 | 20375 | 14520 | 0 | 20581 | 0 | 40015 | 0 | 0 |
| **ctrl-pos2-plt6-1897B-L3** | 17056 | 15305 | 0 | 0 | 6767 |  | 0 |  | 0 | 0 | 0 | 0 |
| ctrl-neg1-pcr2-plt6 | 2 | 1 | 0 | 0 | 0 | 0 | 0 | 0 | 0 | 0 | 0 | 0 |
| ctrl-neg2-pcr2-plt6 | 5 | 4 | 0 | 0 | 0 | 0 | 0 | 0 | 0 | 0 | 0 | 0 |
| ctrl-neg1-pcr1-plt6 | 201 | 25 | 0 | 0 | 0 | 0 | 0 | 0 | 0 | 0 | 0 | 0 |
| ctrl-neg2-pcr1-plt6 | 1975 | 1278 | 0 | 0 | 0 | 0 | 0 | 0 | 0 | 0 | 0 | 0 |
| ctrl-neg1-dna-plt6 | 1464 | 674 | 0 | 0 | 0 | 0 | 134 | 0 | 0 | 0 | 252 | 0 |
| ctrl-neg2-dna-plt6 | 255 | 60 | 0 | 0 | 0 | 0 | 0 | 0 | 0 | 0 | 0 | 0 |
|  |  |  |  |  |  |  |  |  |  |  |  |  |

**Supplementary Table S4:** *Model selection for FEC model*

|  | **Model** | **AIC** |
| --- | --- | --- |
| Model 1 | log(fec_mean + 1) ~ sex + age + weaning + deworming + heart_girth + haemonchus_placei + haemonchus_contortus + cooperia_punctata + trichostrongylus_colubriformis + trichostrongylus_axei + ostertagia_ostertagi + (1 farm_id), data = data) | 1706.9 |
| Model 2 | log(fec_mean + 1) ~ sex + age + weaning + deworming + haemonchus_placei + haemonchus_contortus + cooperia_punctata + trichostrongylus_colubriformis + trichostrongylus_axei + ostertagia_ostertagi + (1 farm_id), data = data) | 1707.3 |
| Model 3 | log(fec_mean + 1) ~ sex + age + weaning + deworming + haemonchus_placei + haemonchus_contortus + cooperia_punctata + trichostrongylus_axei + ostertagia_ostertagi + (1 farm_id), data = data) | 1705.8 |
| Model 4 | log(fec_mean + 1) ~ sex + age + weaning + deworming + haemonchus_placei + haemonchus_contortus + cooperia_punctata + trichostrongylus_axei + (1 farm_id), data = data) | 1704.8 |
| Model 5 | log(fec_mean + 1) ~ sex + age + deworming + haemonchus_placei + haemonchus_contortus + cooperia_punctata + trichostrongylus_axei + (1 farm_id), data = data) | 1703.3 |
| **Final model** | log(fec_mean + 1) ~ sex + age + deworming + haemonchus_placei + haemonchus_contortus + cooperia_punctata + (1 farm_id), data = data) | 1702.3 |

**Supplementary Table S5:** *Model selection for co-infection model*

|  | Model | AIC |
| --- | --- | --- |
| Model 1 | mixed_infection ~ deworming + dewormer_class + sex + age + Combined_breed + management_system + (1 farm_id), data = mdata, family = binomial) | 666.5 |
| **Final Model** | mixed_infection ~ deworming + sex + age + management_system + (1 farm_id), data = data, family = binomial) | 667.5 |
| Interaction model 1 | mixed_infection ~ deworming + sex +age_registration*management_status + (1\|farmer_id), data = mdata, family = binomial | 669.4 |
| Interaction model 2 | mixed_infection ~ deworming*management_status + sex +age_registration + (1\|farmer_id), data = mdata, family = binomial | 669.0 |

*Interactions terms were not statistically significant p > 0.05

**Supplementary Table S6:** *Model selection for heart girth model*

|  | Model | AIC |
| --- | --- | --- |
| Model 1 | heart_girth ~ sex + age + weaning + deworming + haemonchus_placei + haemonchus_contortus + cooperia_punctata + trichostrongylus_axei + trichostrongylus_colubriformis + ostertagia_ostertagi + (1 farm_id), data = data) | 4050.5 |
| Model 2 | heart_girth ~ sex + age + weaning + deworming + haemonchus_placei + haemonchus_contortus + cooperia_punctata + trichostrongylus_colubriformis + ostertagia_ostertagi + (1 farm_id), data = data) | 4051.2 |
| Model 3 | heart_girth ~ sex + age + weaning + deworming + haemonchus_placei + haemonchus_contortus + trichostrongylus_colubriformis + ostertagia_ostertagi + (1 farm_id), data = data) | 4051.8 |
| **Final Model** | heart_girth ~ sex + age + weaning + deworming + haemonchus_placei + trichostrongylus_colubriformis + ostertagia_ostertagi + (1 farm_id), data = data) | 4052.4 |
| Model 5 | heart_girth ~ sex + age + weaning + haemonchus_placei + trichostrongylus_colubriformis + ostertagia_ostertagi + (1 farm_id), data = data) | 4053.0 |
| Model 6 | heart_girth ~ sex + age + haemonchus_placei + trichostrongylus_colubriformis + ostertagia_ostertagi + (1 farm_id), data = data) | 4054.6 |

***Supplementary Table S7:*** *Animal level and management practices of dairy calves on smallholder farms in Nandi County, Kenya (N = 532)*

| Variables | Value | n (%) |
| --- | --- | --- |
| Sex | Female | 303 (57.0) |
|  | Male | 229 (43.0) |
| Weaning status | Not weaned | 289 (54.3) |
|  | Weaned | 243 (45.7) |
| Breed | Holstein Friesian cross | 326 (61.3) |
|  | Ayrshire cross | 193 (36.3) |
|  | Channel island cross | 13 (2.4) |
| Age category(months) | Age 3 - 4 | 174 (32.7) |
|  | Age 5 - 6 | 128 (24.1) |
|  | Age 7 - 9 | 119 (22.4) |
|  | Age 10 - 12 | 111 (20.8) |
| Deworming status | Dewormed | 338 (63.5) |
|  | Not dewormed | 194 (36.5) |
| Class of dewormer | Benzimidazole | 242 (45.5) |
|  | No dewormer | 194 (36.5) |
|  | Imidazothiazoles/macrocyclic lactones | 96 (18.0) |
| Management system | Pasture | 455 (85.5) |
|  | Zero-grazing | 77 (14.5) |

* For the management system, pasture includes also cut-and-carry.

***Supplementary Table S8:*** *Summary Statistics for Fecal Egg Counts (FEC) and Associated Predictor Variables*

|  |  | FEC | | | | | |  |
| --- | --- | --- | --- | --- | --- | --- | --- | --- |
| Variables | n | Mean | Median | SD |  | Q1 | Q3 | |
| Sex |  |  |  |  |  |  |  | |
| Female | 303 | 53.9 | 24.0 | 83.3 |  | 10.0 | 63.3 | |
| Male | 229 | 72.9 | 35.0 | 104.3 |  | 12.5 | 88.0 | |
| Weaning status |  |  |  |  |  |  |  | |
| Not weaned | 289 | 71.0 | 34.5 | 105.7 |  | 12.5 | 82.5 | |
| Weaned | 243 | 51.4 | 21.5 | 74.8 |  | 9.5 | 61.0 | |
| Breed |  |  |  |  |  |  |  | |
| Holstein Friesian cross | 326 | 65.1 | 27.5 | 103.6 |  | 10.5 | 71.5 | |
| Ayrshire cross | 193 | 67.9 | 34.5 | 75.7 |  | 4.5 | 117.0 | |
| Channel Island cross | 13 | 60.0 | 29.0 | 87.6 |  | 11.3 | 69.3 | |
| Age category (months) |  |  |  |  |  |  |  | |
| Age 3 - 4 | 174 | 77.5 | 32.0 | 123.8 |  | 13.5 | 71.3 | |
| Age 5 - 6 | 128 | 56.8 | 31.0 | 71.3 |  | 10.9 | 77.1 | |
| Age 7 - 9 | 119 | 57.0 | 26.5 | 75.8 |  | 10.5 | 66.0 | |
| Age 10 - 12 | 111 | 49.3 | 19.5 | 72.8 |  | 8.8 | 64.0 | |
| Deworming status |  |  |  |  |  |  |  | |
| Dewormed | 338 | 59.0 | 25.0 | 96.0 |  | 8.6 | 65.4 | |
| Not dewormed | 194 | 67.4 | 35.0 | 88.5 |  | 14.1 | 82.6 | |
| Class of dewormer |  |  |  |  |  |  |  | |
| Benzimidazole | 242 | 56.9 | 24.5 | 92.9 |  | 8.5 | 63.9 | |
| Imidazothiazoles/macrocyclic lactones | 96 | 64.2 | 27.5 | 103.7 |  | 9.5 | 69.5 | |
| Management system |  |  |  |  |  |  |  | |
| Pasture | 455 | 63.8 | 28.0 | 96.0 |  | 11.3 | 71.3 | |
| Zero-grazing | 77 | 51.9 | 29.5 | 75.5 |  | 7.0 | 67.0 | |

* The statistics presented include the mean, median, standard deviation (SD), first quartile (Q1) and third quartile (Q3) for each group. Values are rounded to one decimal place.

* For the management system, pasture includes also cut-and-carry.

**Supplementary Table S9:** Univariable analysis of epidemiological factors and Fecal Egg Count (FEC)

| Variables | n | Estimate | 95% Confidence Interval (CI) |
| --- | --- | --- | --- |
| Sex |  |  |  |
| Female | 303 | Ref | - |
| Male | 229 | 0.25 | 0.03 – 0.46 |
| Age (months) |  | **-0.07** | **-0.11 – -0.03** |
| Heart girth |  | **-0.01** | **-0.02 – -0.00** |
| Weaning status |  |  |  |
| Not weaned | 289 | Ref | - |
| Weaned | 243 | **-0.29** | **-0.52 – -0.06** |
| Deworming status |  |  |  |
| Dewormed | 338 | Ref | - |
| Not dewormed | 194 | **0.27** | **0.01 – 0.53** |
| Class of dewormer |  |  |  |
| No dewormers | 194 | Ref | - |
| Benzimidazole | 242 | -0.27 | -0.55 – 0.01 |
| Imidazothiazoles/macrocyclic lactones | 96 | -0.28 | -0.64 – 0.09 |
| Management system |  |  |  |
| Zero grazing | 77 | Ref | - |
| Pasture | 455 | -0.00 | -0.38 – 0.38 |
| Breed |  |  |  |
| Ayrshire and Channel Island cross | 206 | Ref | - |
| Holstein Friesian cross | 326 | -0.01 | -0.23 – 0.22 |

* Channel island cross breed was combined with Ayrshire cross in modelling

* For the management system, pasture includes also cut-and-carry.

**Supplementary Table S10:** Univariable analysis of epidemiological factors and co-infections

| **Variables** | **n** |  | **Odds Ratios** | **95% CI** |
| --- | --- | --- | --- | --- |
| **Sex** |  |  |  |  |
| Female | 303 |  | Ref | - |
| Male | 229 |  | 1.06 | 0.68 – 1.64 |
| **Age (months)** |  |  | 0.98 | 0.90 – 1.05 |
| **Deworming status** |  |  |  |  |
| Dewormed | 338 |  | Ref | - |
| Not dewormed | 194 |  | 0.64 | 0.39 – 1.06 |
| **Class of dewormer** |  |  |  |  |
| Not dewormed | 194 |  | Ref | - |
| Benzimidazole | 242 |  | 1.29 | 0.75– 2.19 |
| Imidazothiazoles/macrocyclic lactones | 96 |  | **2.47** | **1.24 – 4.93** |
| **Management system** |  |  |  |  |
| Zero grazing | 77 |  | Ref | - |
| Pasture | 455 |  | **2.34** | **1.10 – 4.96** |
| **Breed** |  |  |  |  |
| Ayrshire and Channel Island cross | 206 |  | Ref | - |
| Holstein Friesian cross | 326 |  | 0.98 | 0.62 – 1.55 |

*Channel island cross breed was combined with Ayrshire cross

* For the management system, pasture includes also cut-and-carry.

***Supplementary Table S11****: Summary Statistics of heart girth and associated variables*

|  |  | Heart girth | | | | |
| --- | --- | --- | --- | --- | --- | --- |
| Variables | n | Mean | Median | Q1 | Q3 | SD |
| **Sex** |  |  |  |  |  |  |
| Female | 303 | 101.3 | 101 | 92 | 110 | 83.7 |
| Male | 229 | 96.5 | 96 | 87 | 105 | 104.9 |
| **Deworming status** |  |  |  |  |  |  |
| Dewormed | 338 | 98.3 | 98.5 | 88 | 108 | 96.7 |
| Not Dewormed | 194 | 100.7 | 100 | 92 | 108 | 88.7 |
| **Weaning status** |  |  |  |  |  |  |
| Not Weaned | 289 | 95.3 | 96 | 86 | 102 | 106.1 |
| Weaned | 243 | 103.9 | 104 | 96 | 112 | 75.3 |
| **Age category (months)** |  |  |  |  |  |  |
| 3 - 4 | 174 | 89.5 | 89 | 83 | 96 | 125 |
| 4 -6 | 128 | 99.3 | 99.5 | 92 | 106 | 71.3 |
| 6-9 | 119 | 104 | 104 | 98 | 110 | 76.1 |
| 9-12 | 111 | 109 | 106 | 100 | 116 | 73.1 |
| **Management system** |  |  |  |  |  |  |
| Pasture | 455 | 99 | 99 | 89 | 107 | 96.6 |
| Zero-grazing | 77 | 100.2 | 101 | 92 | 110 | 75.5 |
| **Class of dewormer** |  |  |  |  |  |  |
| Benzimidazole | 242 | 98 | 98 | 88 | 107 | 93.3 |
| Imidazothiazoles/macrocyclic lactones | 96 | 99.2 | 100 | 89 | 110 | 105.1 |
| **Breed** |  |  |  |  |  |  |
| Ayrshire cross | 193 | 98.3 | 98 | 88 | 107 | 104.3 |
| Channel Island cross | 13 | 98 | 103.5 | 85.5 | 109 | 78.6 |
| Holstein Friesian cross | 326 | 99.8 | 100 | 91 | 108 | 87.8 |

*The statistics presented include the mean, median, standard deviation (SD), first quartile (Q1) and third quartile (Q3). Values are rounded to one decimal place.

* For the management system, pasture includes also cut-and-carry.

**Supplementary Table S12:** Univariable analysis of heart girth

| Variables | n | Estimate | 95% CI |
| --- | --- | --- | --- |
| **Sex** |  |  |  |
| Female | 303 | Ref | - |
| Male | 229 | **-4.69** | **-7.07 – -2.31** |
| **Age (months)** |  | **2.71** | **2.34 – 3.07** |
| **Weaning status** |  |  |  |
| Not weaned | 289 | Ref | – |
| Weaned | 243 | **8.68** | **6.29 – 11.07** |
| **Deworming status** |  |  |  |
| Dewormed (Ref) | 338 | Ref | – |
| Not dewormed | 194 | 2.34 | -0.42 – 5.09 |
| **Class of dewormer** |  |  |  |
| No dewormers | 194 | Ref | – |
| Benzimidazole | 242 | -2.53 | -5.48 – 0.42 |
| Imidazothiazoles/macrocyclic lactones | 96 | -1.85 | -5.69 – 2.00 |
| **Management system** |  |  |  |
| Zero grazing | 77 | Ref | – |
| Pasture | 455 | -1.28 | -5.19 – 2.64 |
| **Breed** |  |  |  |
| Ayrshire and Channel Island cross | 206 | Ref | – |
| Holstein Friesian cross | 326 | 1.25 | -1.27 – 3.78 |

*Channel island cross breed was combined with Ayrshire cross in modelling

* For the management system, pasture includes also cut-and-carry.
