# Supplementary figures and images for: Species diversity and risk factors of gastrointestinal nematodes in smallholder dairy calves in Kenya
Source: Front Vet Sci. 2025 Aug 12;12:1588350. doi: 10.3389/fvets.2025.1588350 (PMC12379043; doi:10.3389/fvets.2025.1588350)

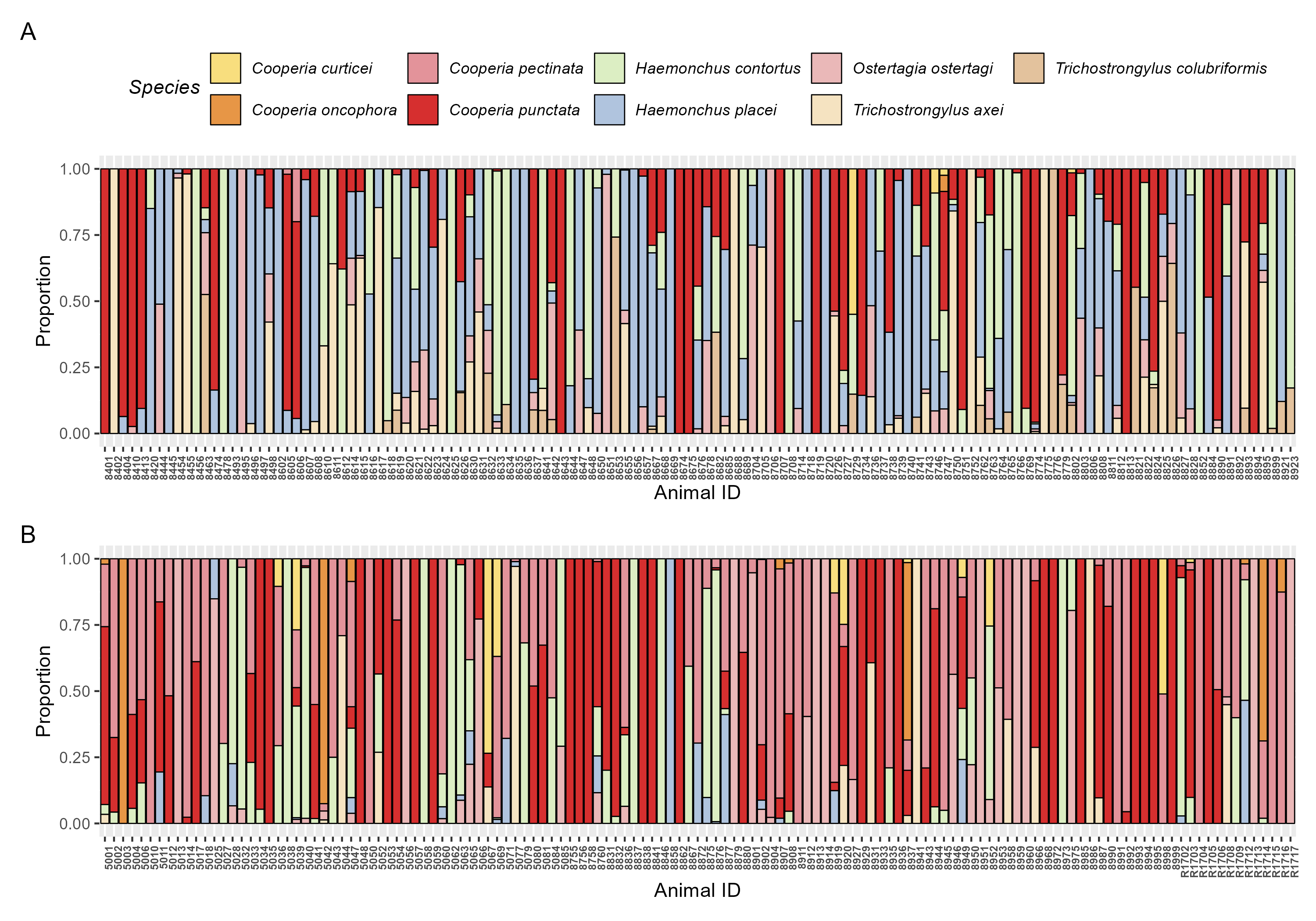

Supplement: Supplementary file 2 [file Image_1.tiff]
